# Idiopathic non-histaminergic acquired angioedema: a case series and discussion of published clinical trials

**DOI:** 10.1186/s13601-017-0164-9

**Published:** 2017-08-31

**Authors:** Martin Christian Bucher, Tatjana Petkovic, Arthur Helbling, Urs Christian Steiner

**Affiliations:** 1 Department of Clinical Immunology, University Hospital Zurich, Gloriastrasse 23, 8091 Zurich, Switzerland; 2 Adverse Drug Reaction-Analysis and Consulting (ADR-AC) GmbH, Bern, Switzerland; 3 Department of Rheumatology, Immunology and Allergology, Inselspital, University Bern, Bern, Switzerland

**Keywords:** Idiopathic angioedema, Mast cell, FcεRI-receptor density, Omalizumab

## Abstract

**Background:**

Idiopathic non-histaminergic acquired angioedema (InH-AAE) is a rare disease for which there are no available laboratory parameters to clearly define the disorder. Therapy is often difficult and various treatment options have been proposed. In this paper, we have evaluated the most effective therapies for InH-AAE on the basis of current literature and report the therapeutic effect of omalizumab in three patients with InH-AAE.

**Methods:**

Literature was searched with a combination of MeSH/EMTREE terms and freetext search for angioedema and therapy/omalizumab in the databases Medline (Ovid), PubMed/Premedline, Embase, Cochrane library and Scopus with no time or language restrictions. In three patients affected by InH-AAE the therapeutic effect of omalizumab was demonstrated by clinical outcome. In one patient the FcεRI receptor density on basophils was monitored under therapy with omalizumab.

**Results:**

From the review of the current literature, 25 out of 286 publications dealing with relevant therapeutic recommendations for InH-AAE were analyzed. Six publications with 98 patients referred to tranexamic acid, of which 27 had a complete, 70 a partial and 1 no response. In three case reports ecallantide showed 2 patients with a complete and 1 a partial response. In four case reports for Icatibant 2 had a complete and 3 a partial response. When evaluated in three reports, C1-INH found complete and partial responses in 2 patients each. One patient had a complete response to progestin. Omalizumab was described in 6 reports with 20 patients, all of whom showed a complete response. All three patients described in our study responded to omalizumab with a complete remission. Density of FcεRI receptors on basophils, monitored in patient 1 on a long-term course of 31 months, decreased from 74,051.61 to a minimal level of 1907 receptors per cell.

**Conclusions:**

Omalizumab seems to be the most effective therapy in InH-AAE. The continuous decrease of FcεRI-receptor density on basophils under therapy with omalizumab along with clinical improvement observed in one patient, could serve as a new approach for further studies to evaluate FcεRI-receptor density as a surrogate marker for therapeutic efficacy and for dosing and determining injection intervals of omalizumab.

*Trial registration* BASEC-Nr. Req-2016-00692. Retrospectively registered 24.11.2016.

**Electronic supplementary material:**

The online version of this article (doi:10.1186/s13601-017-0164-9) contains supplementary material, which is available to authorized users.

## Background

Angioedema (AE) is defined as a localized swelling of the subcutaneous and/or submucosal tissue caused by an increase of vascular permeability, which is induced by vasoactive mediators [1]. Although most often associated with urticaria, angioedema represents its own entity [2, 3]. According to a recent consensus report, angioedemas without urticaria are classified in three hereditary (HAE), and four acquired (AAE) forms [4] (Table 1).

**Table 1:** Forms of Angioedemas without wheals

*Hereditary angioedemas*
HAE with C1-inhibitor deficiency (C1-INH_HAE)
HAE with FXII mutations (FXII-HAE)
HAE of unknown origin (U-HAE)
*Acquired angioedemas*
Idiopathic histaminergic acquired angioedema (IH-AAE)
Idiopathic non-histaminergic acuired angioedema (InH-AAE)
Acquired angioedema related to angiotensin-converting enzyme inhibitor (ACEi-AAE)
Aquired angioedema with C1-inhibitor deficiency (C1-INH-AAE)

Modified from Cicardi et al. [4]

Idiopathic AAE are the most frequently occurring forms of angioedemas [3–5]. Diagnostic laboratory parameters are not yet available for diagnosing and differentiating the two idiopathic acquired forms, Idiopathic histaminergic acquired angioedema (IH-AAE) and Idiopathic non-histaminergic acquired angioedema (InH-AAE) [6]. Diagnosis of IH-AAE is considered if the angioedema responds to antihistamines. The lack of response to antihistamines defines InH-AAE [4, 7].

Therapy of InH-AAE is often challenging. This is reflected by a wide variety of therapeutic options for InH-AAE, such as high dosages of antihistamines (AH), icatibant, ecallantide, C1-INH concentrates, glucocorticosteroids (GCS), omalizumab, and other agents.

In this study we give an overview of the different and most effective therapeutic modalities for InH-AAE according the current literature. We further report the clinical response of omalizumab in three patients affected by InH-AAE. In one patient we monitored the FcεRI-receptor density of basophils during therapy over a period of 31 months.

## Methods

The local Ethical Review Board of Zurich assessed the design of this observational and retrospective study and offered a waiver (Req-2016-00692). The study strictly adhered to the principles of good clinical practice and the ethical standards outlined in the Declaration of Helsinki [8]. All patients were verbally informed and gave their written informed consent for this study.

### Patients

All three patients suffering from angioedemas for years were referred to the out-patient clinic of allergy and clinical immunology. After a detailed allergological and immunological work up, they were diagnosed as InH-AAE. Complement factor C4 level, C1 inhibitor level and C1 inhibitor function were within the normal range. None of them underwent treatment with ACE inhibitors, sartans, gliptins or NSAID.

### Comprehensive review of the literature

Literature was searched with a combination of MeSH/EMTREE terms and freetext search for angioedema and therapy/omalizumab in the databases Medline (Ovid), PubMed/Premedline, Embase, Cochrane library and Scopus with no time or language restrictions. The detailed search protocol is shown in Additional file 1.

### FcεRI receptor density measurement

Peripheral blood mononuclear cells (PBMCs) were isolated from anticoagulant-treated venous blood by density gradient centrifugation using Ficoll Paque (Amersham Pharmacia Biosciences, Uppsala, Sweden). The surface density of FcεRI on donor basophils was determined by means of DAKO QIFIKIT^®^ (Dako-Cytomation, City, Denmark): Freshly isolated PBMCs were resuspended in CellWASH^®^ (BD Bioscience, San Jose, CA, USA) and stained with primary mouse monoclonal anti-FcεRI antibodies (eBioscience, San Diego, CA, USA). Subsequently, goat anti-mouse fluorescein isothiocyanate (FITC) conjugated immunoglobulin F(ab’)_2_ fragments were added to detect bound anti-FcεRI (Dako Denmark, 2008, QIFIKIT^®^ Code K0078). Finally, to identify basophils, staining with anti-CCR3-PE (BioLegend, San Diego, CA, USA) was performed. The antibody-binding capacity of basophils was determined by the mean fluorescent intensity measured by the FACSCanto^®^ (BD Bioscience, San Jose, CA, USA) flow cytometer using a calibration curve set up with beads of known antibody concentration from the DAKO QIFIKIT^®^.

## Results

### Comprehensive review of the literature

From the 499 retrieved publications 213 were duplicates. Of the remaining 286 publications, 25 trials, consisting of case reports, case series and retrospective observational studies with relevant therapeutic recommendations for InH-AAE, were selected and analyzed.

Six case reports or retrospective trials describe the efficacy of tranexamic acid (TA) in InH-AAE. Out of 98 patients, 27 responded completely, 70 partially, and 1 patient showed no response [3, 9–13].

Three case reports showed a complete response in 2 and a partial response in 1 patient treated with ecallantide [14–16]. Therapy with icatibant is described in four case reports. A complete response was shown in 2, and a partial response in 3 patients [14, 17–19].

In three case reports with C1-INH 2 patients had a complete and 2 patients a partial response [14, 19, 20].

In a retrospective study of 50 patients, progestin decreased the frequency of angioedema attacks in 45 women. Twenty of them were affected by idiopathic AE and 80% responded with a partial response [21].

In six reports 20 patients with InH-AAE were treated with omalizumab. All had a complete response [22–27].

Five Individual reports with 1 patient each were described for ciclosporin with no response [28], dapsone [29], fresh frozen plasma [30], rituximab [31] and cannabis [32]—all of them with a complete response (Table 2).

**Table 2:** Comparison of the therapies most often applied in InH-AAE

	Reference	Number of patients	Drug/doses	Response: cr/pr/nr (number of patients)
*Medical influence of coagulation and contact activation pathway*				
Tranexamic acid	[9]	2	1–4 g per day	cr 2
	[10]	1	*NA*	cr 1
	[11]	15	0.5–3 g per day	cr 8/pr 7
	[12]	23	0.5–3 g per day	cr 12/pr 11
	[13]	19	1–3 g per day	cr 4/pr 15
	[3]	38	0.5–3 g per day	pr 37/nr 1
Ecallantide	[15]	1	30 mg (during attacks)	cr 1
	[16]	1	30 mg (during attacks)	cr 1
	[14]	1	*NA*	pr 1
Icatibant	[17]	1	30 mg (during attacks)	cr 1
	[18]	1	30 mg (during attacks)	cr 1
	[14]	1	*NA*	pr 1
	[19]	2	30 mg *(during attack)*	pr 2
C1-INH	[14]	1	1000U twice weekly	cr 1
	[19]	2	*NA*	pr 2
	[20]	1	1000U twice weekly	cr 1
*Medical influence of the hormonal axis*				
Progestin	[21]	20	Various dosages depending the progestin	cr 6/pr 10/nr 4
*Medical influence of IgE antibodies and mast cell (Omalizumab)*				
Omalizumab	[46]	3	300 mg every 3–4 weeks; 375 mg every 2 weeks	Cr 3
	[23]	1	300 mg every 4 weeks initially, then reduced to 300 mg every 8 weeks	cr 1
	[24]	1	300 mg every 4 weeks	cr 1
	[25]	8	300 mg every 4 weeks initially, then reduced to doses and intervals according to symptoms	cr 8
	[27]	2	375 mg every 2 weeks initially, then reduced to 375 mg every 4 weeks	cr 2
	[26]	5	300 mg every 2–4 weeks	cr 5
*Other immunosuppressants or immunomodulatory therapies*				
Dapsone	[29]	1	50 mg per day	cr 1
Ciclosporin	[28]	1	300 mg per day	nr 1
FFP	[30]	1	4 units	cr 1
Rituximab	[31]	1	560 mg (375 mg/m^2^ body surface area) weekly for 4 weeks	cr 1
Cannabis	[32]	1	20 g per month, inhaled 2–3× per week	cr 1

*cr* complete remission, *pr* partial remission, *nr* no response, *NA* not available, *FFP* fresh frozen plasma

### Case reports

#### Patient 1

**Fig. 1:**
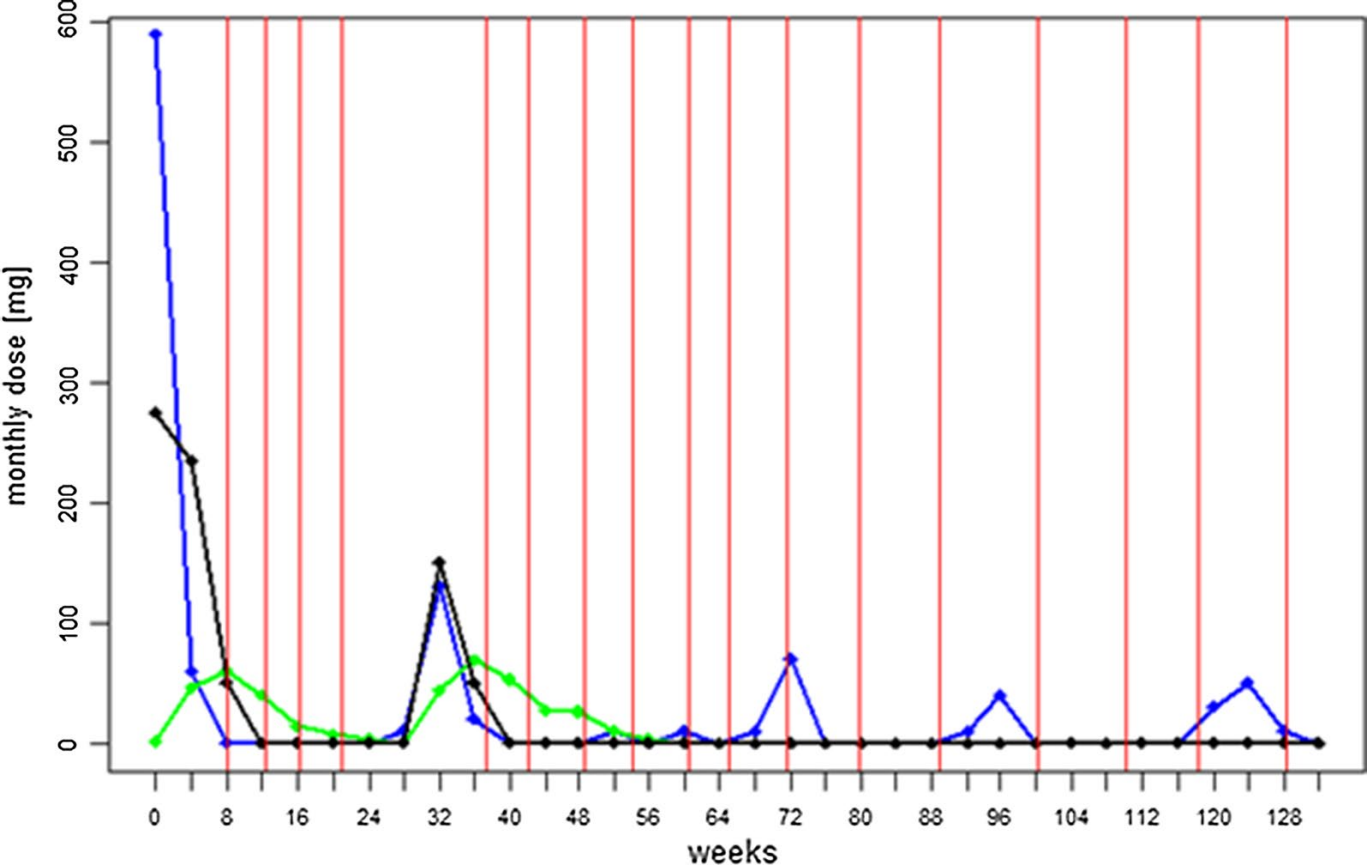
Medication use during treatment with omalizumab in patient 1. *Red solid line* Omalizumab injections; *black solid line* Prednisone intake; *blue solid line* Cetirizine intake; *green solid line* Ketotifen intake. Average doses of AH and oral glucocorticoids were calculated for every month in the past two and a half years

A 67-year-old white female with a 3-year history of recurrent, twice-weekly angioedemas of the lips, tongue and larynx. The clinical response of the angioedemas to the intake of antihistamines (AH) of doses up to three times higher than that normally recommended was not sufficient. Thus, glococorticosteroids (GCS) on a weekly basis were often necessary to control the symptoms. Omalizumab 150 mg was administered subcutaneously in 4-week intervals. After the first injection of omalizumab, facial swellings improved markedly. Treatment with AH and GCS were discontinued after 8 weeks. Omalizumab was paused after the fourth injection. Eleven weeks after stopping therapy, facial angioedemas reoccurred (Fig. 1). Omalizumab 150 mg was resumed and the angioedema disappeared after 1 week. The patient stayed symptom-free. The interval between injections was stretched to every 2 months and the patient stayed symptom-free with only intermittent discrete eyelid swellings.

#### Patient 2

A 46-year-old white male suffering for 9 years from weekly recurrent non-itchy angioedemas on the feet, face and tongue. Due to laryngeal angioedema with airway-occlusion, emergency treatment was necessary in 2013. Suggested triggers were mechanical stress, cold, infections, and NSAIDs. Following a job change with associated mental pressure, angioedemas were not controllable with high doses of AH. Treatment with omalizumab was started, using 300 mg s.c. at monthly intervals. Concomitant AH intake was reduced and finally completely discontinued after 2 weeks. After 1 year of treatment, the dose was reduced to 150 mg and has been given every month since, without recurrence of symptoms.

#### Patient 3

A 27-year-old white female suffering for 8 months from weekly recurrent angioedema of the lips and periorbital region without pruritus. No trigger factors were noticed. Since swelling episodes were not controllable with AH and GCS, doses of 300 mg s.c. of omalizumab were initiated at intervals of every 4 weeks. After the second injection no further angioedema episodes occurred.

### FcεRI-receptor density in patient 1

**Fig. 2:**
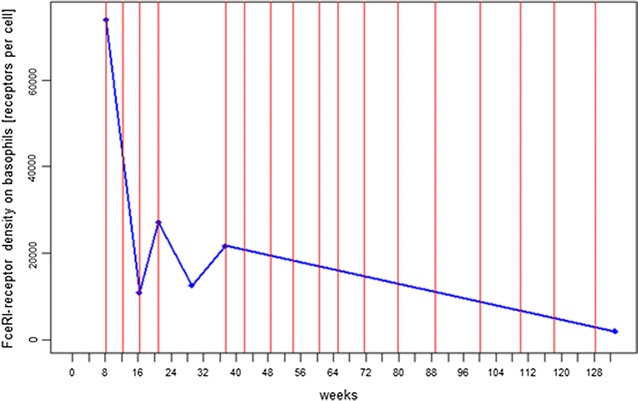
Long-term therapy treatment of density of FcεRI-receptors on basophils in patient 1. *Red solid line* Omalizumab injections; *blue solid line* FcεRI-receptor density. Receptor density decreased substantially during treatment with omalizumab over 31 months from 74,051 receptors per cell before start of treatment, to 1,907 receptors per cell

Density of FcεRI receptor on basophils was monitored in patient 1 (ADR-AC GmbH, Bern, Switzerland). Density decreased remarkably from 74,051.61 to 10,892.93 rpc after the second injection of omalizumab 150 mg. After the 4th injection, after which omalizumab was discontinued, FcεRI-receptor density increased up to 21,666.53 rpc during the 16-week pause. After reintroduction of omalizumab 150 mg at intervals of 4 to 8 weeks over 31 months, the FcεRI-receptor density decreased to a minimal level of 1,907rpc (see Fig. 2).

## Discussion

In 2014 an international working group proposed a classification of angioedemas without wheals [4]. However, characterisation and differentiation of InH-AAE remain difficult and evidence of treatment efficacy is limited to case reports and uncontrolled small case series. Based on the literature, a wide range of different therapies are used for InH-AAE. TA is the most frequently used therapy for Inh-AAE. It inhibits plasminogen activation and therefore limits FXIIa activation and C1-INH consumption by plasmin. This leads to a decrease of bradykinin formation [33, 34]. In the trials described, about one third of the 98 patients had a complete response to TA as prophylaxis for InH-AAE [3, 9–13]. Both ecallantide, a kallikrein receptor antagonist, and C1-INH, a serine protease with inhibitory activities in the complement system and the contact-,coagulation-, and fibrinolytic pathways, reduce the generation of Bradykinin. Ecallantide was effective in all described patients with Inh-AAE for acute attacks. C1-INH was effective for acute attacks and was also valuable for long term prophylaxis [14–16, 19, 20]. Icatibant, a competitive bradykinin B2 receptor antagonist, selectively prevents the effect of bradykinin on the endothelium. Used in acute attacks, it showed a good but short-lived response in most of the patients. Progestin modulates endogenous oestrogen and therefore reduces activation of FXII and the contact activation pathway which leads to a control of angioedema in HAE patients with C1-Inh deficiency [35, 36]. Saule et al. [21] reported favourable experience with progestins to prevent attacks in any type of non-allergic angioedema. Omalizumab, a humanized IgG monoclonal antibody directed against IgE antibodies reduces free IgE levels which results in the downregulation of FcεRI receptors on basophils and mast cells [37, 38]. Given the current state of knowledge, these effects prevent IgG autoantibodies from cross-linking FcεRI receptors, cell activation is suppressed, the threshold of mast cell degranulation is increased, and possible autoreactive IgE antibodies can not longer bind the high affinity receptor [39, 40]. In the described case reports and case series all patients diagnosed with Inh-AAE responded to omalizumab.

With regard to the mode of action of the different medications against InH AAE, the drug either interacts with IgE antibodies and influences the mast cells, as does omalizumab, or it interacts with the coagulation and contact activation pathway, as do tranexamic acid (TA), icatibant, ecallantide, C1-INH and progestin (compare Table 2). This indicates that mast cells and the contact activation pathway are linked by mediators most probably secreted by mast cells. As recently discovered, heparin from activated mast cells induces the contact activation pathway by FXII [41]. These findings beg the conclusion that activated mast cells not only induce histamine induced angioedemas by secreting their mediators.

About 40–50% of patients with chronic spontaneous urticaria (CSU) have episodes of angioedema and a part of them show the symptoms of angioedema without wheals. IH-AAE shares a lot of similarities with CSU. Many patients with CSU remain symptomatic in spite of antihistamine therapy up to four times the approved dose [42, 43]. In these patients ciclosporin and omalizumab are recommended as an addon therapy. When IH-AAE is associated with CSU responding to antihistamines, Inh-AAE should be associated with CSU patients not responding to antihistamines.

All three patients described in this report had full remission after the second injection of omalizumab and remained free of symptoms when omalizumab was given continuously. This is in line with the described case reports and series so far, where all patients treated with omalizumab had a complete response. In patient 1 we were able to document the decrease of receptor density in correlation with clinical response (Figs. 1, 2). The follow-up of FcεRI-receptor density on basophils over 31 months showed an impressive continuous decrease of receptor density when omalizumab was regularly administered and it allowed us to adapt the therapy individually.

## Strength and limitations

In this study a comprehensive overview of the literature of the different treatments in Inh-AAE could be provided. However, a deficiency lies in the fact that the effect of medications interacting with the contact activation pathway in the same patients who responded to omalizumab was not examined. Another downside of the study is that the long term course of FcεRI-receptor density was demonstrated in one patient only.

## Conclusion

InH-AAE shares similarities to patients with CSU which do not respond to antihistamines. Omalizumab is the most promising therapeutic approach for prophylaxis in Inh-AAE. Not only is histamine involved in the formation of angioedemas, but also other mediators of activated mast cells which interact with the contact pathway.

As the clinical course corresponds with FcεRI-receptor density [39], this receptor could be evaluated as a biomarker to monitor the therapeutic effect and adjust the dose of omalizumab for long term therapy.

Better knowledge about how and why mast cells are activated and release their mediators more or less selectively, will enable us to design new and more personalized therapies [44, 45].

## Additional file

**Additional file 1.** The detailed search protocol for Medline.

## Abbreviations

AE: angioedema
C1-INH-HAE: hereditary angioedema with C1 inhibitor deficiency
IH-AAE: idiopathic histaminergic acquired angioedema
InH-AAE: idiopathic non-histaminergic acquired angioedema
AH: antihistamines
GCS: glucocorticosteroids
PBMCs: peripheral blood mononuclear cells
rpc: receptor per cell

## References

[1] Kaplan AP, Greaves MW (2005). Angioedema. J Am Acad Dermatol.

[2] Aberer W (2014). Angioedema is not just ‘deep urticaria’ but an entity of its own. Allergy.

[3] Mansi M, Zanichelli A, Coerezza A, Suffritti C, Wu MA, Vacchini R, Stieber C, Cichon S, Cicardi M (2015). Presentation, diagnosis and treatment of angioedema without wheals: a retrospective analysis of a cohort of 1058 patients. J Intern Med.

[4] Cicardi M, Aberer W, Banerji A, Bas M, Bernstein JA, Bork K, Caballero T, Farkas H, Grumach A, Kaplan AP (2014). Classification, diagnosis, and approach to treatment for angioedema: consensus report from the Hereditary Angioedema International Working Group. Allergy.

[5] Zingale LC, Beltrami L, Zanichelli A, Maggioni L, Pappalardo E, Cicardi B, Cicardi M (2006). Angioedema without urticaria: a large clinical survey. CMAJ.

[6] Farkas H, Veszeli N, Kajdácsi E, Cervenak L, Varga L (2016). “Nuts and bolts” of laboratory evaluation of angioedema. Clin Rev Allergy Immunol.

[7] Faisant C, Boccon-Gibod I, Mansard C, Dumestre Perard C, Pralong P, Chatain C, Deroux A, Bouillet L (2016). Idiopathic histaminergic angioedema without wheals: a case series of 31 patients. Clin Exp Immunol.

[8] World Medical Association (2014). Declaration of Helsinki ethical principles for medical research involving human subjects. J Am Coll Dent.

[9] Thompson RA, Felix-Davies DD (1978). Response of ‘idiopathic’ recurrent angioneurotic oedema to tranexamic acid. BMJ.

[10] Hakansson OM (1988). Menstruation-related angioedema treated with tranexamic acid. Acta Obstet Gynecol Scand.

[11] Cicardi M, Bergamaschini L, Zingale LC, Gioffre D, Agostoni A (1999). Idiopathic nonhistaminergic angioedema. Am J Med.

[12] Du-Thanh A, Raison-Peyron N, Drouet C, Guillot B (2010). Efficacy of tranexamic acid in sporadic idiopathic bradykinin angioedema. Allergy.

[13] Wintenberger C, Boccon-Gibod I, Launay D, Fain O, Kanny G, Jeandel PY, Martin L, Gompel A, Bouillet L (2014). Tranexamic acid as maintenance treatment for non-histaminergic angioedema: analysis of efficacy and safety in 37 patients. Clin Exp Immunol.

[14] Stahl MC, Harris CK, Matto S, Bernstein JA (2014). Idiopathic nonhistaminergic angioedema successfully treated with ecallantide, icatibant, and C1 esterase inhibitor replacement. J Allergy Clin Immunol Pract.

[15] Berry A, Firszt R (2013). Successful treatment of idiopathic angioedema with ecallantide. J Allergy Clin Immunol Pract.

[16] Dy TB, Rasheed M, Parikh P, Bernstein L (2013). Resolution of an acute attack of idiopathic angioedema with ecallantide. Ann Allergy Asthma Immunol.

[17] Del Corso I, Puxeddu I, Sardano E, Geraci S, Breggia M, Rocchi V, Migliorini P (2012). Treatment of idiopathic nonhistaminergic angioedema with bradykinin B2 receptor antagonist icatibant. Ann Allergy Asthma Immunol.

[18] Montinaro V, Loizzo G, Zito A, Castellano G, Gesualdo L (2013). Successful treatment of a facial attack of angioedema with icatibant in a patient with idiopathic angioedema. Am J Emerg Med.

[19] Shroba J, Hanson J, Portnoy J (2015). Current treatment options for idiopathic angioedema. Ann Allergy Asthma Immunol.

[20] Gravante C, Carucci L, Bova M, Petraroli A, Genovese A, Marone G (2016). Prophylactic treatment with plasma-derived C1 inhibitor in idiopathic non-histaminergic angioedema. Pediatr Allergy Immunol.

[21] Saule C, Boccon-Gibod I, Fain O, Kanny G, Plu-Bureau G, Martin L, Launay D, Bouillet L, Gompel A (2013). Benefits of progestin contraception in non-allergic angioedema. Clin Exp Allergy.

[22] Sands MF, Blume JW, Schwartz SA (2007). Successful treatment of 3 patients with recurrent idiopathic angioedema with omalizumab. J Allergy Clin Immunol.

[23] von Websky A, Reich K, Steinkraus V, Breuer K (2013). Complete remission of severe chronic recurrent angioedema of unknown cause with omalizumab. J Dtsch Dermatol Ges.

[24] Ozturk AB, Kocaturk E (2014). Omalizumab in recurring larynx angioedema: a case report. Asia Pac Allergy.

[25] Azofra J, Díaz C, Antépara I, Jaúregui I, Soriano A, Ferrer M (2015). Positive response to omalizumab in patients with acquired idiopathic nonhistaminergic angioedema. Ann Allergy Asthma Immunol.

[26] Faisant C, Du Thanh A, Mansard C, Deroux A, Boccon-Gibod I, Bouillet L (2016). Idiopathic non-histaminergic angioedema: successful treatment with omalizumab in five patients. J Clin Immunol.

[27] Munoz JP, Casado AF, Taboada AC, Campos Munoz L, Bran EL (2016). Successful treatment of refractory idiopathic angio-oedema with omalizumab: review of the literature and function of IgE in angio-oedema. Clin Exp Dermatol.

[28] Şener O, Bolu E, Akyol S (2005). Cyclosporine A in the treatment of chronic idiopathic angioedema: a case report. Gulhane Med J.

[29] Gonzalez P, Soriano V, Caballero T, Niveiro E (2005). Idiopatic angioedema treated with dapsone. Allergol Immunopathol.

[30] Franzen D, Ursprung T, Wuthrich B, Reber A (2006). Idiopathic non-histaminergic angio-oedema after routine extubation successfully treated with fresh frozen plasma. Anaesthesia.

[31] Ghazan-Shahi S, Ellis AK (2011). Severe steroid-dependent idiopathic angioedema with response to rituximab. Ann Allergy Asthma Immunol.

[32] Frenkel A, Roy-Shapira A, Evgeni B, Leonid K, Borer A, Klein M (2015). Life threatening idiopathic recurrent angioedema responding to cannabis. Case Rep Immunol.

[33] Tengborn L, Blomback M, Berntorp E (2015). Tranexamic acid—an old drug still going strong and making a revival. Thromb Res.

[34] Kaplan AP, Joseph K (2016). Complement, Kinins, and hereditary angioedema: mechanisms of Plasma Instability when C1 Inhibitor is Absent. Clin Rev Allergy Immunol.

[35] Citarella F, Misiti S, Felici A, Farsetti A, Pontecorvi A, Fantoni A (1996). Estrogen induction and contact phase activation of human factor XII. Steroids.

[36] Bork K, Fischer B, Dewald G (2003). Recurrent episodes of skin angioedema and severe attacks of abdominal pain induced by oral contraceptives or hormone replacement therapy. Am J Med.

[37] MacGlashan DW, Bochner BS, Adelman DC, Jardieu PM, Togias A, McKenzie-White J, Sterbinsky SA, Hamilton RG, Lichtenstein LM (1997). Down-regulation of Fc(epsilon)RI expression on human basophils during in vivo treatment of atopic patients with anti-IgE antibody. J Immunol.

[38] Beck LA, Marcotte GV, MacGlashan D, Togias A, Saini S (2004). Omalizumab-induced reductions in mast cell Fce psilon RI expression and function. J Allergy Clin Immunol.

[39] Metz M, Staubach P, Bauer A, Brehler R, Gericke J, Kangas M, Ashton-Chess J, Jarvis P, Georgiou P, Canvin J (2017). Clinical efficacy of omalizumab in chronic spontaneous urticaria is associated with a reduction of FcepsilonRI-positive cells in the skin. Theranostics.

[40] Kaplan AP, Gimenez-Arnau AM, Saini SS (2017). Mechanisms of action that contribute to efficacy of omalizumab in chronic spontaneous urticaria. Allergy.

[41] Oschatz C, Maas C, Lecher B, Jansen T, Bjorkqvist J, Tradler T, Sedlmeier R, Burfeind P, Cichon S, Hammerschmidt S (2011). Mast cells increase vascular permeability by heparin-initiated bradykinin formation in vivo. Immunity.

[42] Maurer M, Weller K, Bindslev-Jensen C, Gimenez-Arnau A, Bousquet PJ, Bousquet J, Canonica GW, Church MK, Godse KV, Grattan CE (2011). Unmet clinical needs in chronic spontaneous urticaria. A GA(2)LEN task force report. Allergy.

[43] Guillen-Aguinaga S, Jauregui Presa I, Aguinaga-Ontoso E, Guillen-Grima F, Ferrer M (2016). Updosing nonsedating antihistamines in patients with chronic spontaneous urticaria: a systematic review and meta-analysis. Br J Dermatol.

[44] Theoharides TC, Kempuraj D, Tagen M, Conti P, Kalogeromitros D (2007). Differential release of mast cell mediators and the pathogenesis of inflammation. Immunol Rev.

[45] Harvima IT, Levi-Schaffer F, Draber P, Friedman S, Polakovicova I, Gibbs BF, Blank U, Nilsson G, Maurer M (2014). Molecular targets on mast cells and basophils for novel therapies. J Allergy Clin Immunol.

