# Supplementary material for: Idiopathic non-histaminergic acquired angioedema: a case series and discussion of published clinical trials
Source: Clin Transl Allergy. 2017 Aug 31;7:27. doi: 10.1186/s13601-017-0164-9 (PMC5577688; doi:10.1186/s13601-017-0164-9)
Supplement: Supplementary file 1 — Additional file 1. The detailed search protocol for Medline. [file 13601_2017_164_MOESM1_ESM.docx]

**Search Strategy:**

| **#** | **Searches** | **Results** |
| --- | --- | --- |
| 1 | (idiopathic adj6 (angioedema* or angioedema* or angio-edema* or angio-oedema* or "giant urtica" or "giant urticaria" or "urtica gigantea" or "urticaria edematosa" or "urticaria oedematosa")).ti,ab. | 135 |
| 2 | (Angioedema/ or (("acute circumscribed" or "acute essential" or neurogenic or quincke* or wandering) adj3 (edema or oedema)).ti,ab. or (angioneurotic adj3 (edema or oedema or swelling or syndrome)).ti,ab.) and (idiopathic adj6 (edema or oedema or syndrome or swelling)).ti,ab. | 33 |
| 3 | 1 or 2 | 154 |
| 4 | (therap* or treat* or random* or dosage or (clinical adj3 trial)).ti,ab. or exp clinical trial/ or exp Therapeutics/ or Random Allocation/ or ("drug therapy" or therapy or "therapeutic use").sh. | 8158715 |
| 5 | 3 and 4 | 100 |
| 6 | Omalizumab/ or ("hu 901" or hu90 or "monoclonal antibody E 25" or "monoclonal antibody E25" or olizumab or Xolair).ti,ab. or (icatibant or firazyr or "WIN 65365 " or "Hoechst 140" or "hoe 140" or hoe140 or "je 049" or je049).ti,ab. | 2806 |
| 7 | 5 and 6 | 16 |
| 8 | 5 not 7 | 84 |
